# In-depth phenotyping of lymphoblastoid cells suggests selective cellular vulnerability in Marinesco-Sjögren syndrome

**DOI:** 10.18632/oncotarget.19663

**Published:** 2017-07-28

**Authors:** Laxmikanth Kollipara, Stephan Buchkremer, José Andrés González Coraspe, Denisa Hathazi, Jan Senderek, Joachim Weis, René P. Zahedi, Andreas Roos

**Affiliations:** ^1^ Leibniz-Institut für Analytische Wissenschaften–ISAS –e.V., 44227 Dortmund, Germany; ^2^ Institute of Neuropathology, University Hospital Aachen, RWTH Aachen, 5274 Aachen, Germany; ^3^ Friedrich-Baur-Institute, Medical Faculty, Ludwig-Maximilians-University, 80336 Munich, Germany; ^4^ The John Walton Muscular Dystrophy Research Centre, MRC Centre for Neuromuscular Diseases, Newcastle University, Newcastle upon Tyne, NE1 3BZ, UK

**Keywords:** Marinesco-Sjögren syndrome, woozy mouse, SIL1, ataxin-10, chaperonopathy

## Abstract

SIL1 is a ubiquitous protein of the Endoplasmic Reticulum (ER) acting as a co-chaperone for the ER-resident chaperone, BiP. Recessive mutations of the corresponding gene lead to vulnerability of skeletal muscle and central nervous system in man (Marinesco-Sjögren syndrome; MSS) and mouse. However, it is still unclear how loss of ubiquitous SIL1 leads to selective vulnerability of the nervous system and skeletal muscle whereas other cells and organs are protected from clinical manifestations. In this study we aimed to disentangle proteins participating in selective vulnerability of SIL1-deficient cells and tissues: morphological examination of MSS patient-derived lymphoblastoid cells revealed altered organelle structures (ER, nucleus and mitochondria) thus showing subclinical vulnerability. To correlate structural perturbations with biochemical changes and to identify proteins potentially preventing phenotypical manifestation, proteomic studies have been carried out. Results of proteomic profiling are in line with the morphological findings and show affection of nuclear, mitochondrial and cytoskeletal proteins as well as of such responsible for cellular viability. Moreover, expression patterns of proteins known to be involved in neuromuscular disorders or in development and function of the nervous system were altered. Paradigmatic findings were confirmed by immunohistochemistry of splenic lymphocytes and the cerebellum of SIL1-deficient mice. Ataxin-10, identified with increased abundance in our proteome profile, is necessary for the neuronal survival but also controls muscle fiber apoptosis, thus declaring this protein as a plausible candidate for selective tissue vulnerability. Our combined results provide first insights into the molecular causes of selective cell and tissue vulnerability defining the MSS phenotype.

## INTRODUCTION

Marinesco-Sjögren syndrome (MSS; MIM 248800) is an autosomal recessive neurodegenerative disorder caused by mutations in the *SIL1* gene [1–3]. Almost all SIL1 mutations reported are expected to lead to loss of the corresponding protein SIL1 [4, 5]. SIL1 acts as a nucleotide exchange factor for the main chaperone of the endoplasmic reticulum, BiP [6, 7]. MSS-patients present with cerebellar ataxia, severe progressive myopathy and bilateral cataracts as well as mental impairment of varying degree [4]. A gene-trapped *Sil1* mutant mouse model also shows cerebellar atrophy - due to Purkinje-cell degeneration - and a progressive myopathy [8–11]. Both, the human and the mouse *SIL1/Sil1* genes are ubiquitously expressed. MSS is believed to be caused by a disturbed SIL1-BiP-machinery and hence malfunction of ER-processes related to BiP function [7]. However, it is still unknown why functional loss of a ubiquitously expressed protein causes a selective vulnerability of certain tissues, especially the nervous system and skeletal muscle. Surprisingly, loss of SIL1 does not affect the ability of mouse B cells and of human EBV-transformed lymphoblastoid cells (LCs) to assemble and secrete antibodies [12], the best characterized substrates of BiP [13–15]. Although other functional studies suggest that nucleotide exchange factors are required for efficient antibody assembly and secretion [16, 17], no evidence for compensatory activation of another molecular chaperone system has been obtained thus far [12]. Ultrastructural studies of MSS-patient-derived skin fibroblasts revealed morphological alterations [5], suggesting subclinical vulnerability. For these reasons, we explored whether *(i)* MSS-patient derived peripheral blood cells also present with morphological perturbations indicative of subclinical vulnerability and *(ii)* aimed to gain insights into potential antagonizing mechanisms preventing phenotypical manifestation of SIL1-deficiency. To achieve these goals, we used Epstein-Barr Virus (EBV)-transformed LCs derived from four different genetically proven MSS-patients [4], and carried out transmission electron microscopic together with comprehensive proteomic profiling studies as well as further immunoblotting and –histochemistry studies to verify the proteomic findings and to obtain deeper insights into selective organ vulnerability.

## RESULTS AND DISCUSSION

### TEM findings of MSS-lymphoblastoid cell lines

Recalling morphological alteration in MSS-patient derived fibroblasts [5] as a cellular population clinically not affected by SIL1-loss, we investigated whether SIL1-deficient LCs also present with ultra-structural perturbations. Transmission electron microscopic (TEM) studies revealed regular organelle structures in LCs derived from healthy controls (Figure 2A, 2B). In contrast, patient-derived LCs recapitulate findings obtained in vulnerable cells and tissues such as SIL1-depleted HEK293 cells, woozy-mouse derived Purkinje cells (PCs) and muscle fibres as well as MSS-patient muscle fibres: widened ER structures and enlarged spaces between inner and outer nuclear membrane (Figure 2C–2E) as well as vacuoles (Figure 2C, 2D, 2E, 2P), some of which were filled with membranous material indicating proteolysis (Figure 2H, 2L, 2S) [10, 18] were found. Electron-denseautophagic material in the cytoplasm was also occasionally detectable in SIL1-affected LCs (Figure 2J, 2K, 2M). Moreover, enlarged and disorganized mitochondria were observed (Figure 2F–2M). This finding suggests a functional connection between the SIL1-BiP chaperone system and mitochondrial homeostasis, possibly through structural organelle cross-talk [19, 20]. Some MSS-LCs presented with nuclear invaginations, sometimes filled with disorganized mitochondria (Figure 2M, 2N). Moreover, irregular accumulations of electron-dense material in the nucleoplasm could also be observed (Figure 2E). Some nuclei of MSS-LCs presented with a minor degree of chromatin condensation at the nuclear membrane (Figure 2C, 2D), abnormal nuclear lobulation (Figure 2Q–2R) or nuclear segmentation (Figure 2S, 2T). Nuclear damage has also been observed in human and mouse SIL1-deficient muscles [4, 21, 22] and was correlated to alterations of nuclear proteins [10]. More recently, we have shown perturbed nuclear structures and altered expression of proteins of the nuclear envelope in SIL1-depleted HEK293 cells [23]. Since BiP is enriched within the nuclear envelope [10, 24, 25] and similar morphological alterations were described in cells deficient for the BiP binding partner SigR1 [18], the BiP chaperone system is obviously important for the control of nuclear integrity [10, 23].

**Figure 1:**
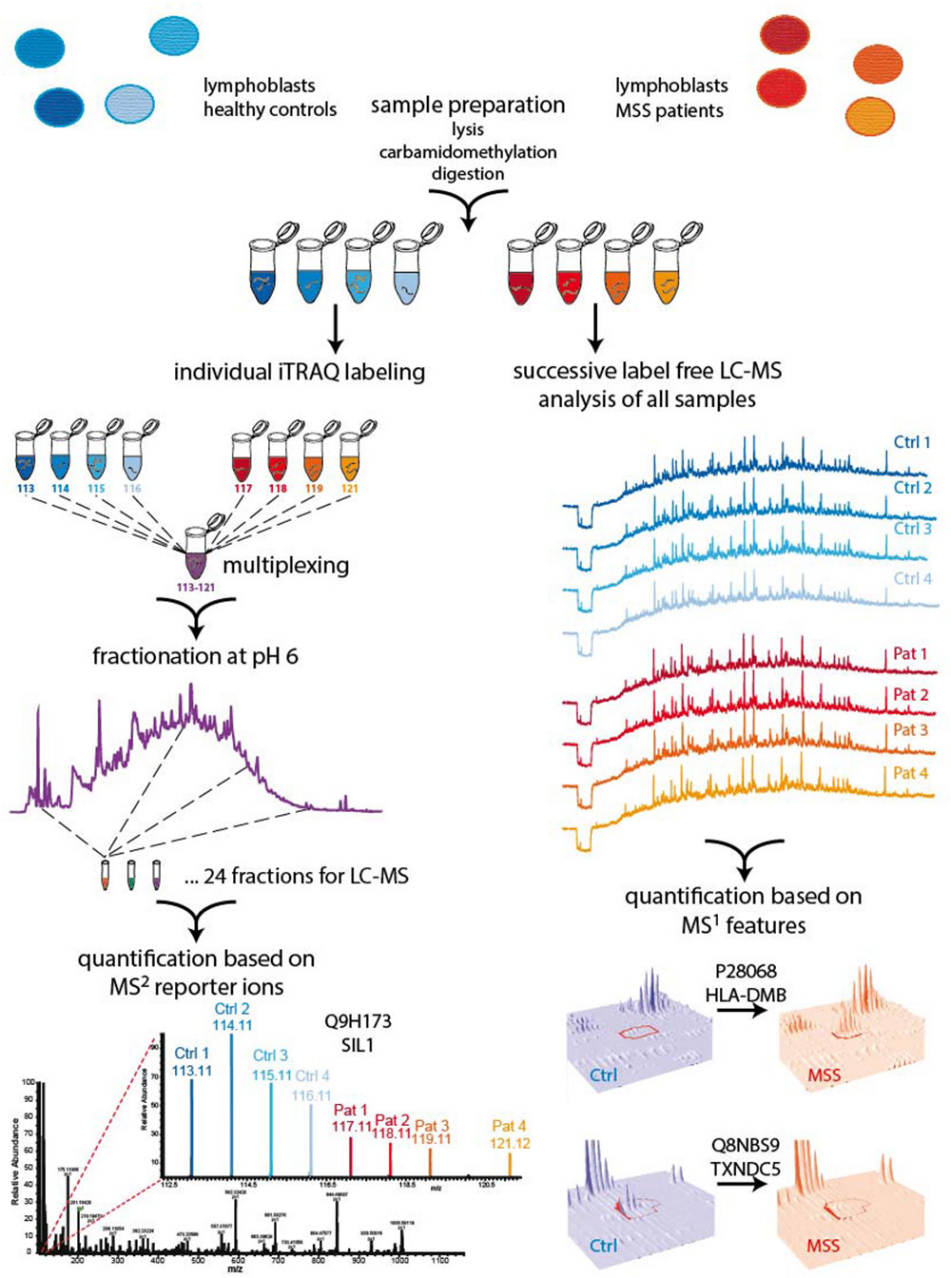
Two-pronged proteomic workflow using (i) label free quantification for more accurate ratio determination between MSS and control samples and (ii) iTRAQ-based quantification to obtain a deeper proteome coverage Cells were lysed, proteins carbamidomethylated and digested using trypsin. Generated peptide samples were either iTRAQ 8plex labeled and multiplexed or analysed individually for label free LC-MS analysis followed by quantification with Progenesis and Peptide Shaker. To obtain a deeper coverage the multiplexed iTRAQ sample was fractionated by reversed phase chromatography at pH 6.0 and fractions were analysed by LC-MS, followed by reporter ion quantification using Proteome Discoverer.

**Figure 2:**
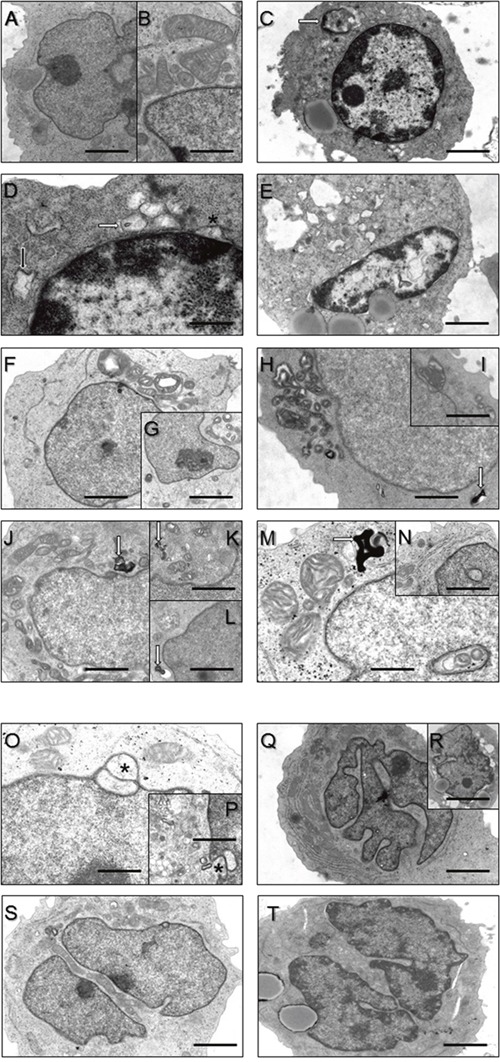
Ultrastructural findings in control lymphoblastoid cells (LC) (A, B) and LC derived from MSS patients (C-V) **(A, B)** Regular organelle structures in LC derived from healthy probands. Scale bars in A = 5 μm; in B = 2 μm. **(C)** Hyperchromasia of the nucleus and cytoplasmic accumulation of electron-dense material (white arrow) in a LC derived from a MSS patient. Scale bar = 7.5 μm. **(D)** Higher magnification of **(C)** emphasizing widened rough ER (black arrow) and outfoldings of the lifted-off nuclear envelope (asterisk) as well as accumulation of vesicular structures partially filled with electron-dense material. Scale bar = 1 μm. **(E)** Another representative MSS patient- derived LC presenting with hyperchromasia of the nucleus, increased outfoldings of the lifted-off nuclear envelope, irregular accumulation of electron-dense material within the nucleoplasm and large cytosolic vacuoles. Scale bar = 7.5 μm. **(F-I)** proliferation of disorganized mitochondria. Cell depicted in the inset G in addtion to disorganized mitochondria also shows (electron-lucent) vacuole. Scale bars in F = 5 μm; in H = 4 μm; in G, I = 10 μm. Occasionally accumulation of myelin-like autophagic material (white arrows in **H**, **J-M)**. Scale bars in H, J = 4 μm; in K, L = 7 μm; in M = 2 μm. **(M, N)** show nuclei with “cytoplasmic halos” partially filled with abnormal mitochondria **(M)**. Scale bar in N = 6 μm. **(O, P)** Nuclear envelope protrusions (asterisks) and considerable vesicular proliferations. Scale bars in O = 3 μm; in K = 7 μm. **(Q, R)** Irregularly shaped/lobulated and **(S, T)** splitted/fragmented nuclei. Scale bars in Q = 7 μm; in S, T = 5 μm.

Taken together, our morphological findings shows that presence of functional SIL1 is required for the maintenance of cellular organelles in both, MSS-vulnerable and non-vulnerable cells and tissues. Based on our findings, we speculated that *(i)* antagonizing molecular strategies, preventing non-vulnerable cells from clinically relevant pathology in MSS are efficiently activated and that *(ii)* SIL1-deficiency affects proteins particularly important for the proper function of neurons and skeletal muscle.

### Proteome profiling of MSS-lymphoblastoid cell lines

**Figure 3:**
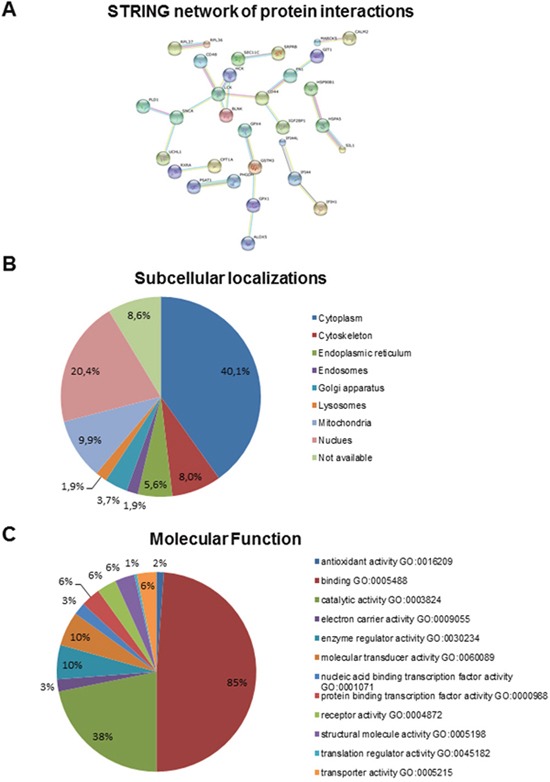
**(A)** STRING-analysis of interaction networks of proteins altered in SIL1-deficient LCs revealed connective groups of eleven, four, three (twice) and two (quintuply) proteins, respectively. **(B, C)** Summary of predicted gene product function and location using gene ontology terms. Gene ontology (GO) terms for annotated human proteins were extracted, if present, from the GO database and sorted into the immediate subcategories for molecular function, cellular component and biological process. The GO subcategory and percentage relative to the total number of extracted terms is indicated.

We compared the proteomic signature of LCs derived from four different male MSS individuals (genetically and biochemically proven for loss of functional SIL1 [4]) with four cell lines derived from healthy donors matched for age and sex. Using liquid chromatography-mass spectrometry (LC-MS) based quantitative proteomics based on isobaric tagging for relative and absolute quantification [26] (iTRAQ 8plex) as well as label free protein quantification (Figure 1) we could quantify 4,389 and 2,756 proteins (≥ 2 unique peptides, 1% FDR), respectively (Supplementary Table 1). Compared to the detection and quantification rates of other proteomic studies using LCs, our work provides the most comprehensive proteome profile of human lymphoblasts published so far [27–29]. Our studies revealed that 162 proteins (nearly 4% of all quantified proteins) showed altered abundances in MSS-LCs of which 59 proteins were up- and 103 proteins were decreased. SIL1, previously described as a low abundant ER-resident co-chaperone [30, 31] was among the downregulated proteins (Figure 1), thus confirming the sensitivity of our proteomic approach. Using STRING software [32], for 31 out of the 162 proteins a known interaction could be detected (Figure 3A) with high confidence, indicating a functional interplay of factors modulated by SIL1-deficiency.

Gene product functions and locations were predicted and categorized using gene ontology terms and sorted into the immediate subcategories for molecular function and cellular component. The GO subcategory and percentage relative to the total number of extracted terms is indicated in Figure 3B and 3C. More detailed information concerning protein functions were obtained from the respective information listed in UniProtKB; data which are not derived from this resource are marked by their references within this text. Our studies revealed that proteins localized in the Golgi-ER network (including proteins of the secretory pathway), in the plasma membrane, in mitochondria, in the cytoplasm (cytoskeleton), the nucleus, and proteins shuttling between the two latter compartments were predominantly altered (Figure 3B, Supplementary Table 2). Involvement of proteins localized to these subcellular compartments is in agreement with the morphological findings (see above). Functionally, proteins required for maintenance of the cytoskeleton, cellular differentiation, antigen representation, immune response and pro- and anti-apoptotic processes are regulated. Remarkably, several of the altered proteins altered are major determinants of the proper function of nervous system and of skeletal muscle (see below).

#### SIL1 deficiency modulates control of cellular fitness

**Figure 4:**
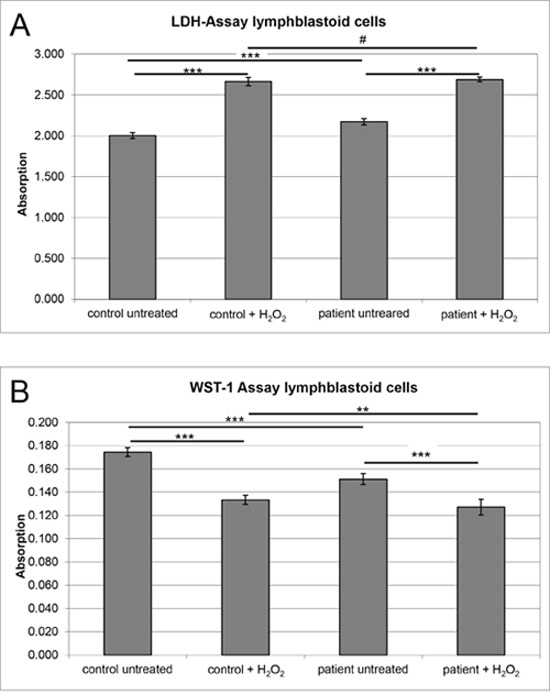
Analyses of cellular fitness in SIL1-deficient LCs and control cells MSS-derived LCs and control LCs included in the MS studies were pooled for the experiments. **(A)** Diagram shows absorption reflecting cytotoxicity upon SIL1-deficiency in MSS-derived LCs in comparison to control cells derived from healthy donors. LDH released from control cells was considered as a baseline (Y-axis). SIL1 deficient LCs show a ∼ 10% increased cytotoxicity compared to the controls. H_2_O_2_ treated cells showed the expected considerable cytotoxic increase confirming functionality of this assay. After stressing no significant difference in cytotoxicity could be detected between the both pooled cell lines. **(B)** Diagram shows absorption reflecting proliferation upon SIL1-deficiency in MSS-derived LCs in comparison to control cells derived from healthy donors. WST-1 conversion to the red soluble formazan in control cells was considered as a baseline (Y-axis). SIL1 deficient LCs show a ∼ 23% decreased proliferation rate compared to the controls. H_2_O_2_ treated cells showed the expected considerable viability reduction confirming functionality of this assay. After stressing, SIL1-deficient cells solely reveal a minor reduction in proliferation compared to the controls and compared to the experiment using the untreated cells.

**Figure 5:**
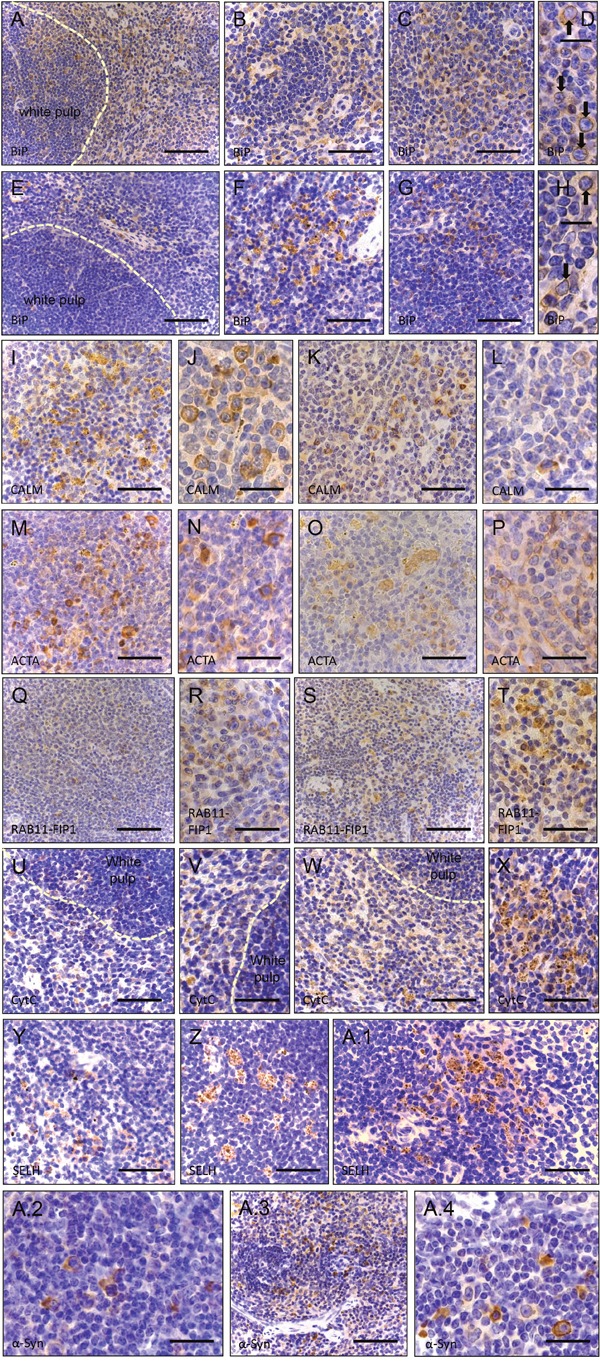
Immunohistochemistry of paraffin sections of paraformaldehyde-fixed spleen specimens of 26-week old control (wt) and of *Sil1* mutant mice **(A-D)** BiP immunoreactivity of the ER and of the nuclear envelope (black arrows in **D)** in wt animals. Scale bars A = 100 μm; in B, C = 110 μm; in D = 50 μm **(B-D** = red pulp). Whereas overall BiP immunoreactivity of the ER is reduced in *Sil1* deficient animals, focal enrichment of BiP within the nuclear envelope (black arrows in **H**) is similar to the control animals. Scale bars E = 100 μm; in F, G = 110 μm; in H = 50 μm (E-H = red pulp). Calmodulin (CALM) immunoreactivity of the red pulp in wild-type animals **(I-J)** and reduced immunoreactivity of the red pulp in *Sil1* mutant animals **(K, L)**. Scale bars in I, K = 110 μm; in J, L = 75 μm. Cells of white and red pulp show stronger actin (ACTA) immunoreactivity in wt **(M, N)** than in *Sil1* mutant animals **(O, P)**. Scale bars in M, O = 110 μm; in N, P = 75 μm. **(Q, R)** Less intense RAB11-FIP1 immunoreactive structures in the red pulp of wt animals compared to in *Sil1* mutant animals **(S, T)**. Scale bars in Q, S = 100 μm; in R, T = 140 μm. **(U, V)** Cytochrome C (CytC) reduction in wt animals compared to *Sil1* mutant littermates **(W, X)**. Scale bars in U, W = 110 μm; in V, X = 140 μm. Analogous reduction of Selenoprotein H (SELH) immunoreactivity in wt **(Y)** in comparison to SIL1 deficient splenic cells **(Z, A.1)**. Scale bar in Y = 100 μm; in Z, A.1 = 110 μm. α-synuclein (α-Syn) staining is less prominent in wt splenic cells **(A.2)** compared to cells in *Sil1* mutant mice **(A.3, A.4)**. Scale bars in A.2, A.4 = 75 μm; in A.3 = 150 μm.

**Figure 6:**
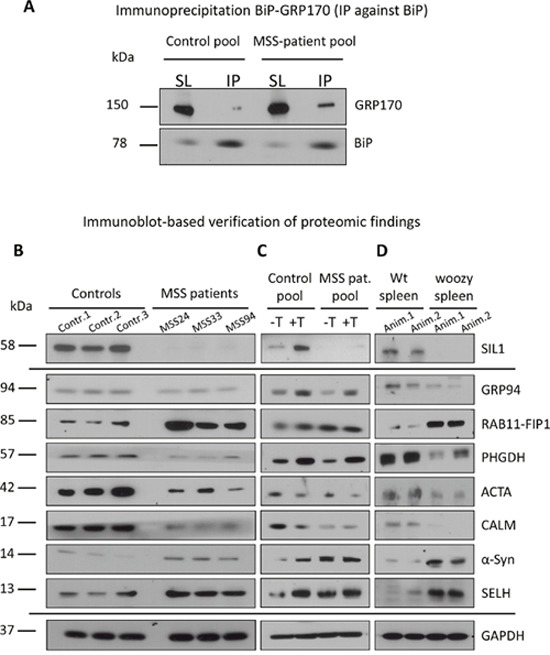
Immunoblot studies on SIL1 mutant lymphoblastoid cells and tissue **(A)** BiP-immunoprecipitation in control and MSS-patient derived lymphoblastoid cells and subsequent analyses of amont of BiP-bound GRP170 reveals a higher amount of BiP-associated GRP170 in the patient cell pool compared to the controls. **(B)** Immunoblot-based verification of protoemic findings for paradigmatic proteins (SIL1, GRP94, RAB11-FIP1, PHGDH, ACTA, CALM, α-Syn & SELH) utilizing an idependent batch of control and MSS-patient derived LCs. Investigation confirmed a decrease for SIL1, GRP94, PHGDH, ACTA, & CALM as well as an increase for RAB11-FIP1,α-Syn & SELH. GAPDH has been used to demonstrate equal protein loading. **(C)** Investigation of ER-stress responsiveness of the above mentioned paradigmatic proteins confirmed the known up-regulation of SIL1 and GRP94 upon presence of thapsigargin-induced ER-stress and moreover revealed a similar effect for RAB11-FIP11 (but not further elevated in patient cells upon thapsigargin-treatment), PHGDH and SELH. Increase of α-Syn upon thapsigargin-treatment is most likely in agreement with the build-up of protein aggregates. GAPDH has been used to demonstrate equal protein loading. **(D)** Immunoblot-based verification of altered abundance addressed via immunohistochemistry for the panel of paradigmatic proteins shown in Figure 5 utilizing spleen of two idependent wildtype and woozy animals, respectively. Investigation confirmed a decrease for SIL1, GRP94, PHGDH, ACTA, & CALM as well as an increase for RAB11-FIP1,α-Syn & SELH. GAPDH has been used to demonstrate equal protein loading.

**Figure 7:**
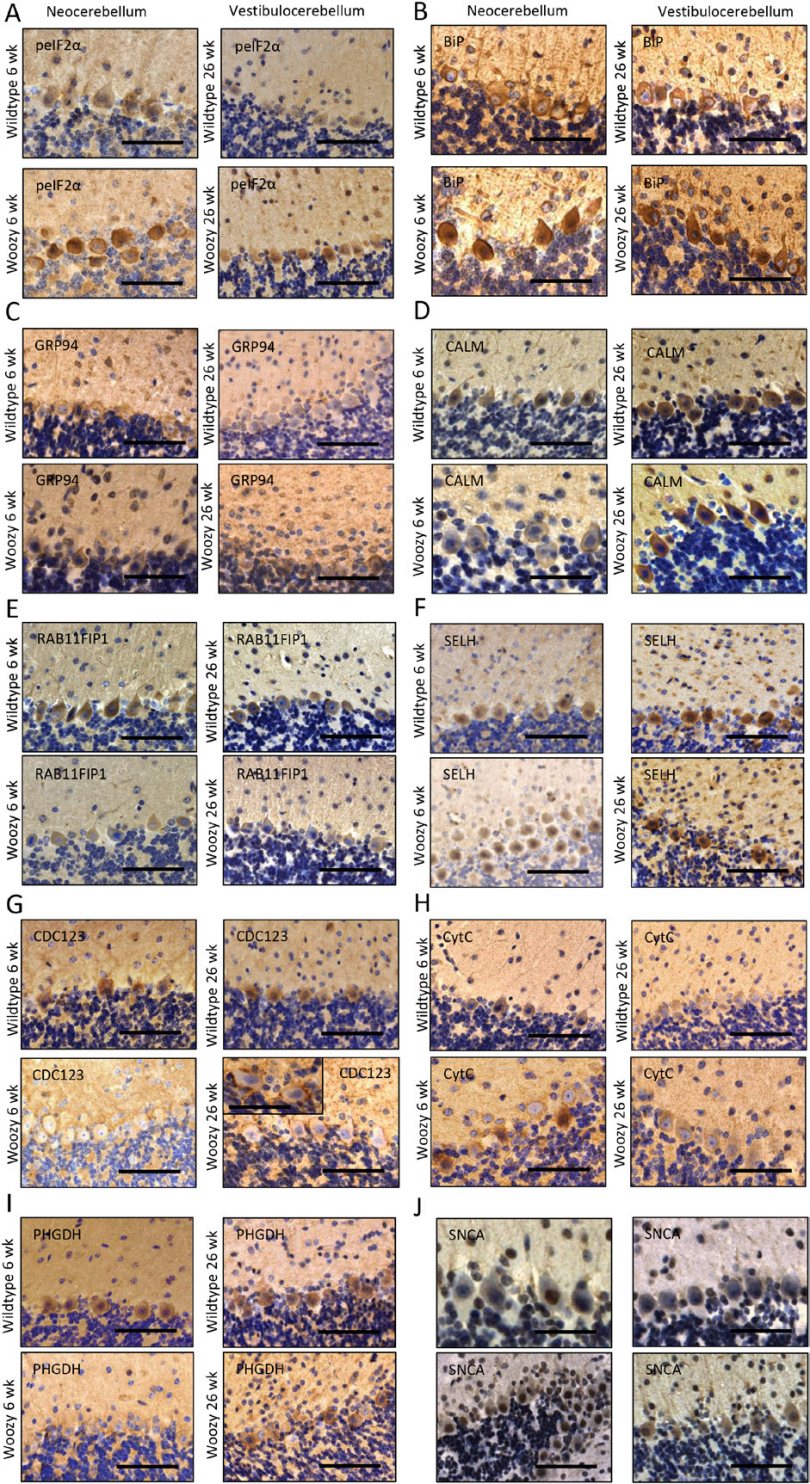
Immunohistochemistry of paraffin sections of paraformaldehyde-fixed cerebellar specimens of 6-week and 26-week old control (wt) and of *Sil1* mutant mice (woozy) **(A)** phospho-eIF2α immunoreactivity of the cerebellar brain areas. Scale bars = in neocerebellum 50 μm, in vestibulocerebellum 75 μm. **(B)** BiP immunoreactivity of the ER and of the nuclear envelope. Scale bars = in neocerebellum 45 μm, in vestibulocerebellum 60 μm. Both UPR-related proteins show increased immunoreactivity in Sil1-defienct PCs of neo- and vestibulocerebellum (lower panels) compared to wt littermates (upper panels). **(C)** PCs of woozy animals (lower panels) show no stronger GRP94 immunoreactivity, neither in the neo- nor in the vestibulocerebellum compared to wt animals (upper panels). Scale bars = in neocerebellum 50 μm, in vestibulocerebellum 60 μm. **(D)** CALM reduction in the degenerating PCs of the neo- but not in the vestibulocerebellum (lower panels) of woozy animals compared to similar brain areas of wt littermates (upper panels). Scale bars = in neo- and vestibulocerebellum of wildtype animals 60 μm, in neo- and vestibulocerebellum of woozy animals 50 μm. **(E)** Compared to wt animals (upper panel), decreased RAB11-FIP1 immunoreactivity of Sil1-deficient PCs in both, the neo- and the vestibulocerebellum (lower panels) is detectable. Scale bars = in neocerebellum 60 μm, in vestibulocerebellum 75 μm. **(F)** SELH immunostaining shows no changes in protein abundance in Sil1-deficient PC populations (lower panels) compared to controls (upper panels). Scale bars = in neo- and vestibulocerebellum 75 μm. **(G)** CDC123 shows decreased immunoreactivity in cell bodies of Sil1-deficient PCs but is increased at the plasma membrane of same cells (lower panels) compared with PCs of wt animals (upper panels). This peripheral enrichment is more apparent in disease-resistant PCs of the vestibulocerebellum of woozy animals. Scale bars = in neocerebellum and vestibulocerebellum 65 μm, in inset 40 μm. **(H)** CytC is increased in degenerating PCs of the neocerebellum of woozy animals but not in surviving PCs of the vestibulocerebellum of woozy mice (lower panels) compared to respective control mice (upper panel). Scale bar = in neo- and vestibulocerebellum of wildtype animals 65 μm, in neo- and vestibulocerebellum of woozy animals 50 μm. **(I)** PCs of Sil1-deficient neocerebellum show decreased immunoreactivity compared to PCs of Sil1-deficient vestibulocerebellum (lower panel) and PCs of similar brain areas of wt controls (upper panel). Scale bar = in neocerebellum of wildtype animal 60 μm, in vestibulocerebellum of wildtype animal 70 μm, in neo- and vestibulocerebellum of woozy animals 75 μm. **(J)** SNCA/α-synuclein shows increased immunoreactivity in PC nuclei in the neocerebellum of woozy animals but not in surviving PCs of the vestibulocerebellum of wt mice (lower panels). Sclale bar = in neocerebellum of wildtype animal 35 μm, in vestibulocerebellum of wildtype animal 40 μm, in neocerebellum of woozy animal 75 μm, in vestibulocerebellum of woozy animal 60 μm.

As a consequence of a defective SIL1-BiP machinery, it is expected that ER stress cannot be alleviated properly, ultimately resulting in cell death. However, results of viability assays focusing on proliferation (WST-1 assay) and cytotoxicity (LDH assay) did not indicate a severe impairment of cellular fitness (Figure 4). This indicates the activation of competing pro-survival mechanisms in LCs that might be less active or efficient in clinically affected cell populations. The most plausible explanation would be activation of rescue chaperone systems. However, in contrast, we found decreased levels of BiP and GRP94 (Figures 5 and 6), two components of the SIL1-complex [7] as well as of ASNS, C19orf10, CHORDC1, FKBP11 and SDF2L1, which are known to be involved in activation of UPR as a cellular stress defense mechanism. Notably, BiP also showed reduced immunoreactivity in spleen derived from *Sil1* mutant animals (Figure 5). These combined data confirm the findings of a recent study published by Ichhaporia and co-workers [12]. Such a downregulation of proteins involved in UPR has also been described in LCs derived from spinocerebellar ataxia subtype 17 (SCA17) patients [29]. However, we hypothesized that loss of functional SIL1 affecting proper BiP function can be (partially) compensated by forced binding of the alternative co-chaperone GRP170 to BiP and thus performed respective immunoprecipitation studies in control and patient-derived LCs. Indeed, results of this experiment reveal a forced binding of GRP170 to BiP in the absence of SIL1 (Figure 6A). After confirming our proteomic findings for paradigmatic proteins via immunoblotting (Figure 6B), we studied the vulnerability of these proteins against additional ER-stress burden and showed that GRP94, PHGDH and SELH increase under (further) stress conditions in both, control and MSS-LCs (Figure 6C). Notably, for the first time an ER-stress dependent alteration of protein abundance has been shown for SELH and PHGDH based on our findings (Figure 6C). In contrast to the findings in LCs (Figures 5 and 6), the immunohistochemical study of ER-stress related proteins is indicative for activation of UPR in SIL1-deficient PCs (Figure 7A and 7B). This finding confirms the previous findings reported by Zhao and co-workers [8, 9]. However, like in MSS-LCs, GRP94 did not show increased abundance in PCs of woozy cerebella (Figure 7C) suggesting that this UPR proteins is not involved in UPR upon *Sil1* mutation in this cellular population. Our proteomic profiling also revealed a decrease of proteins involved in the ubiquitin proteasome pathway including KCMF1, KEAP1, PSMB5, TBL1XR1, UBAP2, UBE2E2 and UCHL1, once more highlighting that other defense mechanisms must be activated in order to maintain proper cellular functions and survival. Notably, we observed a decrease of proteins involved in co-translational translocation and processing of nascent proteins into the ER-lumen (SRPRB, RPL36 and 37, SEC11C). Extenuated protein translation is implied by decrease of ribosomal proteins (NIP7, OGFOD1, RPL36 and 37), pre-mRNA processing/splicing factors (FAM98A, PRPF38B, RBM22, TXNL4A and ZNF598) and a factor involved in ER-Golgi mediated protein modification, STT3A. Decrease of these proteins reflects a cellular strategy antagonizing ER-overload with unfolded proteins (which is shown by an elevation of intracellular levels of proteins that are secreted under healthy conditions (FN1, GC1, ITIH2 and LGALS1)).

Morphological abnormalities (e.g. occasional electron-dense deposits probably corresponding to protein aggregates) and some of the proteome profiling results display altered viability of MSS-LCs: we observed *(i)* down-regulation of the ER-resident anti-apoptotic protein TXNDC5 [33], *(ii)* altered expression of proteins involved in proliferation (BCL3, BOD1L1, CDC123, IFI44, IFI44L [34], PUM2 and RXR) and *(iii)* lowered expression of a protein with cytoprotective functions, HMOX1. *(iv)* MPST (decreased) acts as an antioxidant and is an important producer of the (neuro)cellular protectant hydrogen sulfide. *(v)* GCSAM (elevated) negatively regulates lymphocyte motility. A critical role of Ca^2+^/Calmodulin-dependent mechanisms in controlling cell survival or death decision of lymphoblasts was already demonstrated for Morbus Alzheimer [35] for which we recently demonstrated a role of SIL1 in neuronal survival [36]. The proteomic signature of MSS-LCs revealed *(vi)* a simultaneous decrease of CALM, BiP, EDF1, MARCKS, and RAP1GDS1, proteins decisive for Ca^2+^/Calmodulin-dependent mechanisms. As viability assays showed no major effects in SIL1-deficient LCs, compensatory mechanisms are likely to exist protecting LCs but probably being less efficient in clinically affected tissues. In order to address this assumption, immunohistochemical studies of spleen and cerebellum derived from woozy mice were carried out: whereas the vulnerable PCs of woozy neocerebella presented with decreased CDC123 level in the cell body and increased level at the plasma membrane only in a minority of PCs, this pro-survival protein is also decreased in the cell bodies but considerable increased at the plasma membrane of these PC bodies within woozy vestibulocerebella (Figure 7G). Hence, a protective function of CDC123 expression and localization in MSS-pathology can be postulated. Calmodulin is also decreased in non-vulnerable spleen and the vulnerable PCs of the neocerebellum but not in the disease-resistant PCs of the vestibulocerebellum of woozy (Figures 5, 6D, 7D). Within the cerebellum calmodulin is found predominantly in PCs, with lower levels in granule cells and interneurons and cerebellar calmodulin expression is significantly reduced in cerebellar ataxic Staggerer and Pogo mice [37, 38].

As proper vesicular transport is essential for neuronal maintenance and survival, increase of related proteins such as RAB11-FIP which regulates endosomal trafficking [39] in MSS-LCs but not in both, vulnerable and non-vulnerable PCs of woozy (Figures 6B, 6C, 7E), makes a neuroprotective role of this protein in MSS-pathogenesis unlikely.

Defense against oxidative stress burden is not only displayed by increase in mitochondrial proteins (see above), but also by elevation of CYB5R3, GPX1, HAAO and SELH. Dissimilar the MSS-LCs, SELH seems not to be changed in degenerating and surviving PCs of woozy (Figure 7F). However, as the immunoreactivity against SELH within the vestibulocerebellum is general higher, this finding does not necessarily suggest that this redox-related protein is not involved protection against oxidative stress and thus in death or survival of SIL1-deficient PCs.

#### SIL1-deficiency alters mitochondrial protein composition

ER and mitochondria form physical interactions involved in the regulation of mitochondrial energetics and apoptotic signaling cascades [19]. Mitochondrial dysfunction induced by protein misfolding in the ER is involved in various neurodegenerative and neuromuscular disorders [40] and deficiency of SIL1/Sil1 causes mitochondrial alterations in patients, mice and *in vitro* models [10, 23]. Mitochondrial pathology observed in MSS-derived LCs by transmission electron microscopy (Figure 2) is reflected by an increase of mitochondrial proteins promoting apoptosis, including cytochrome C (CYCS) and PYCARD [41]. By confirming our proteomic findings, we detected increased CYCS in spleen as a tissue not vulnerable in MSS (Figure 5) and in degenerating neocerebellar PCs but not in surviving vestibulocerebellar PCs of woozy (Figure 7H) showing that cytochrome C increase is involved in the death-survival response of neurons in SIL1/Sil1 pathophysiology. Degeneration of cerebellar Purkinje neurons is known to be associated with a marked increase in immunoreactivity of cytochrome C [42]. In addition, a negative regulator of mitochondrial fusion called VAT1 is increased in MSS-LCs. This result might reflect mitochondrial fragmentation, a cellular process leading to apoptosis (Figure 2) and thus also supports our PYCARD and CYCS findings. Additionally, the decrease of mitochondrial proteins such as CHCHD4, CPT1A, ISCU, MP68, MTRF1L, SCO2, SLC25A4, TOM1L2 and TXNRD2 suggests mitochondrial dysfunction. The up-regulation of the mitochondrial proteins ACADSB, CYB5R3, GPX4, HIBCH and NCF4 is in agreement with the activation of pro-survival/mitoprotective pathways in SIL1 deficient LCs [43, 44].

#### SIL1-deficiency and organelle disturbances affecting the cytoskeleton

Although the ER can form networks independently of cytoskeletal structures, the integrity and distribution of this compartment also influences cytoskeletal composition [20, 45]. Remodeling of the cytoskeleton affecting both actin filaments and microtubules upon loss of functional SIL1 in LCs is further supported by the down-regulation of DPYSL2, FLNA, FLNB, GIT1, MAP7D1, TNIK and TUBB2A as well as up-regulation of AIM1, ARHGAP15, CAPG, CNN2, CNN3, EPB41L2, EVL, IQGAP2, MYH10, PDLIM1, RHOC, and SEPT1. Remarkably, for some of the cytoskeletal proteins affected by SIL1-deficiency it is known that they are essential for CNS function and maintenance (for instance DPYSL2 & GLG1). Consequently, alterations of these proteins in SIL1 deficient nervous tissue seem to contribute to neurodegeneration in MSS.

The Golgi apparatus is involved in cytoskeleton via linker proteins and is thus involved in the axoplasmic flow of fast-moving macromolecules and the orthograde, retrograde, and transsynaptic transport of exogenous ligands. Therefore, neurons are considered particularly susceptible to impaired Golgi function. [45]. We identified decreased levels of the Golgi apparatus membrane protein GLG1, the Golgi organization protein TJAP1, the trans-Golgi network protein sorting factor GGA1 as well as of SLC30A7, a protein controlling zinc homeostasis of this compartment. A previous study focused on the molecular basis of Golgi fragmentation and linked these proteins to vulnerability of motor neurons similar to the vulnerability of motor neurons induced by microtubule-depolymerization in Amyotrophic Lateral Sclerosis (ALS) [46], a disease for which a neuroprotective role of SIL1 was pointed out [23, 47].

#### SIL1 deficiency and expression of proteins involved in immune response

Although the decrease of proteins involved in immune response (BLNK, BST2, CD48, CD82, ICAM2 and SEMA7A) might imply a vulnerability to infectious diseases in SIL1-deficient patients, this does not hold true for MSS patients. In MSS-derived LCs, we found an increase of CD44, HCK, HLA-DMB, -DOA, -DOB and MYO1G, proteins known to be involved in the immune response. One might speculate that this increase reflects acellular compensatory strategy which is concordant with the activation of cellular pro-survival mechanisms and the decrease of apoptotic proteins such as DAP1, DDX47 [48], H3.1 [49], NCOA5 [50] and TIA-1. Moreover, increase of further proteins in MSS-derived LCs is in accordance with the activation of pro-survival strategies: during cellular stress, increased IGF2BP1 stabilizes target mRNAs that are recruited to stress granules, including CD44 transcripts and increase of CD44, an antigen important for lymphocyte activation, which was also concomitantly regulated. Increased LCK plays a role in the IL2 receptor-linked signaling pathway that controls the T-cell proliferative response, whereas IL16 which induces T-lymphocyte expression of IL2 receptor was also found to be increased.

#### Several altered proteins are required in neuronal development and function and are involved in neurological diseases

A critical role of SIL1 in neuronal survival and function was illustrated by PC death in both woozy mice and MSS patients, by intellectual deficits and occasionally motor neuronopathy in MSS patients [2, 4, 8, 9, 51], and its role in model systems for Amyotrophic Lateral Sclerosis (ALS) [47] and Alzheimer disease (AD) [36, 52]. Proteomic profiling of MSS-derived LCs disclosed alterations of cellular organelles and related proteins that may render neurons and glia cells sensitive to a wide variety of injuries [53, 54]. Moreover, activation of competitive apoptotic and pro-survival mechanisms which can be potentially more or less effective in neuronal populations or skeletal muscle than in other tissues can be deduced from our proteomic data (see above). Finally, several proteins which are necessary for the proper function of the skeletal muscle are affected by loss of SIL1 in LCs. SMAD4, decreased in MSS-derived LCs, plays a central role in hypertrophy of skeletal muscle [55], and TIA1 which is also down-regulated, is mutated in Welander distal myopathy (WDM). WDM, an autosomal dominant disorder, is characterized by distal muscle weakness with myopathic changes including prominent rimmed vacuoles [56].

It is well known that under such circumstances α-synuclein becomes misfolded and neurotoxic and causes further mitochondrial dysfunction contributing to the neurodegeneration in Parkinson's disease (PD) [57]. We found that α-synuclein expression is increased in MSS-derived LCs and SIL1-deficient mouse spleen (see below). Thus, we assume that the increase of α-synuclein in SIL1 deficient neuronal populations contributes to the neurodegeneration in MSS. Interestingly, study of α-synuclein in PCs revealed no aggregates but a nuclear enrichment in a minority of these cells in the neocerebellum of wildtype animals and in the majority of PCs of woozy animals (Figure 7J). Nuclear enrichment of α-synuclein could not be detected in the PCs of the non-vulnerable vestibulocerebellum. Because α-synuclein promotes neurotoxicity in nuclei, we postulate its involved in the death-survival response of neurons in SIL1 pathophysiology. Interestingly. Byrne and co-workers reported on a SIL1-related MSS-case with marked bradykinesia, hypomimia and difficulties initiating movements before the ataxia typically associated with MSS became prominent [51].

Results of our proteomic study allow direct insights into the role of SIL1 cerebellar ataxia, a hallmark of MSS: the proteome profile of MSS-derived LCs revealed decrease of PHGDH, a protein that was linked to a phenotype including cerebellar ataxia, cataracts and mild psychomotor retardation which is very similar to MSS [58]. Our immunohistological findings revealed that PHGDH is decreased in spleen and in the degenerating PC population of the neocerebellum but not in PCs of the non-vulnerable vestibulocerebellum in woozy (Figure 7I). As loss of this protein causes ataxia and mental impairment, a particular role of PHGDH in neuronal homeostasis is free of doubt and thus its down-regulation is more detrimental in PCs than in LCs or spleen.

As MSS-patients present with varying degree of cognitive dysfunction [4], it seems plausible that the extent of altered protein expression controls severity of cognitive impairment: The protein kinase BRAF and LZTFL1, both altered in MSS-LCs, play a roles in hippocampal neurons [59, 60], a cellular population sensitive to changes in the amount of SIL1 [52].

#### Ataxin-10 as a key modulator of selective vulnerability in SIL1 deficient tissue

**Figure 8:**
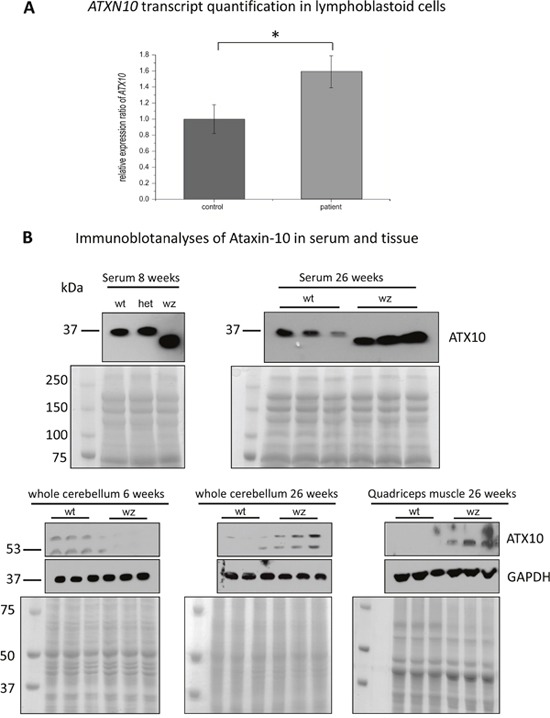

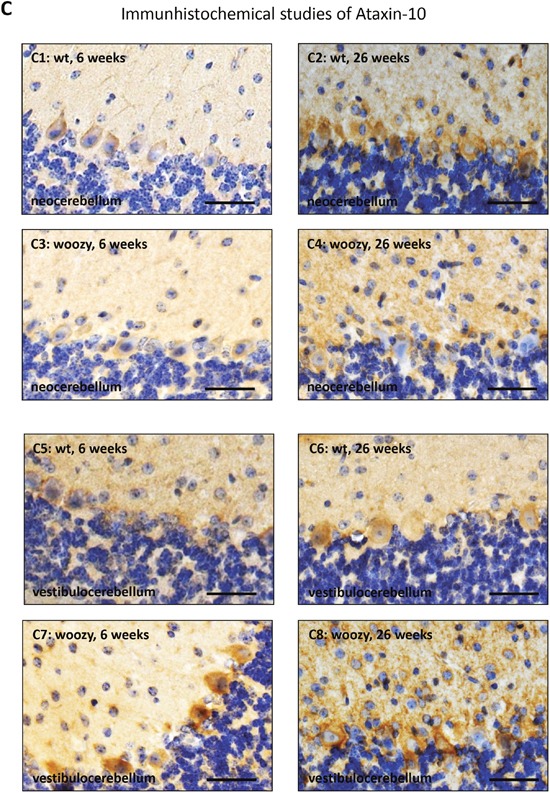

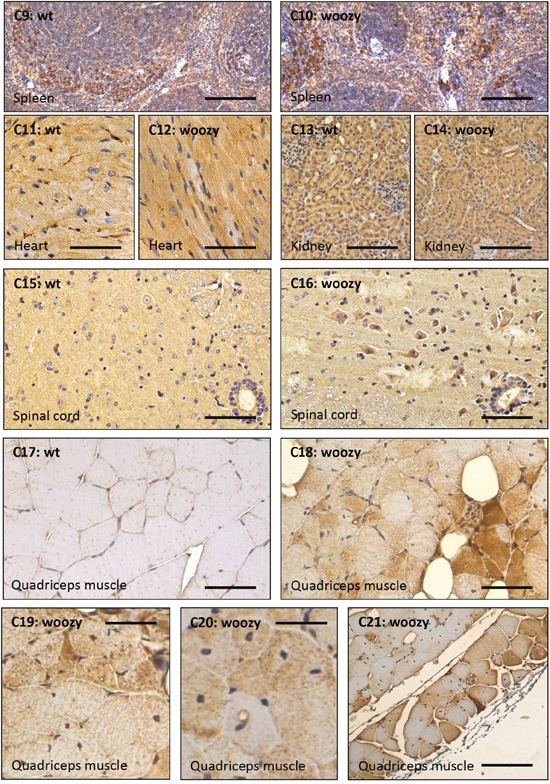
Further studies of ataxin-10 transcript and protein abundance **(A)** Targeted transcript studies of *ATXN10* utilizing LCs derived from healthy controls and MSS-patients (same batch of cells used for the proteomic studies) revealed a 1.6-fold increase in patient-derived LCs. **(B)** Immunoblot-based examination of ATNX10 level in sera derived from woozy animals and wildtype littermates (as well as one heterozygous animal). Whereas in sera of the wildtype littermates and in the heterozygous animal a band at 37 kDa could consistantly be detected, in woozy sera the utilized antibody detected a band with a molecular weight lower than 37 kDa. Coomasie blue staining has been carried out to demonstrate equal protein loading. Further immunoblot-based investigation of ataxin-10 protein level in whole cerebellar protein lysates derived from 6- and 26-week old woozy and control animals shows a reduced netto abundance in 6-week old animals when PC degeneration in initiated (lower left panel). In contrast, in whole cerebellar protein lysates derived from 26-week old animals (surviving PC population in the vestibulocerebellum), increased abundance can be detected (lower panel in the middle). In both, the 6- and the 26-week old animals an addional band (of higher molecular weight; approx. 65 kDa) showing similar regulation of protein abundance can be detected. Coomasie blue staining has been carried out to demonstrate equal protein loading. Investigation of whole quadriceps muscle protein lysates derived from 26-week old woozy and control animals revealed increased ataxin-10 abundance in the diseased muscle of the *Sil1* mutant animals (lower right panel). Here, only one band at the predicted ataxin-10 size of 53 kDa could be detected. Coomasie blue staining has been carried out to demonstrate equal protein loading. **(C)** Immunohistochemistry of paraffin sections of paraformaldehyde-fixed specimens of 6-week and 26-week old control (wt) and of Sil1 mutant mice (woozy) revealed reduced ataxin-10 abundance in the vulnerable neocerebellum at the age of 6- and 26-week old woozy animals compared to the wildtype littermates (C1-C4) and increased ataxin-10 abundance in the non-vulnerable vestibulocerebellum at the age of 6- and 26-week old woozy animals compared to the wildtype littermates (C5-C8). Increased immunoreactivity can be observed in PCs and the neuropil. Sclale bars = 40 μm. No obvious changes in ataxin-10 abundance can be detected in the spleen, the heart and the kidney of *Sil1* mutant animals compared to the wildtype controls (C9-C14; 26-week old). Scale bars: in C9 and C10 = 50 μm, in C11 and C12 = 60 μm, in C13 and C14 = 40 μm. Increased immunoreactivity in motoneurons of *Sil1* mutant animals compared to the wildtype controls (C15 and C16; 26-week old). Scale bars = 50 μm. Increased ataxin-10 immunoreactivity can be observed in diseased and degenerating muscle fibres of *Sil1* mutant animals compared to the wildtype controls (C17-C21; 26-week old). Scale bars: in C17 and C18 = 65 μm, in C19 = 4 μm, in C20 = 30 μm, in C21 = 100 μm.

Ataxin-10 (ATXN10) is a cytoplasmic protein and a member of the ataxin protein family which is ubiquitously expressed in nervous tissue. Loss of ATXN10 in primary neuronal cells causes increased apoptosis of cerebellar neurons. Overexpression of ATXN10 in PC12 cells induced neurite extension and enhanced neuronal differentiation [61]. Controversial to the beneficial functions in neuronal cells, it has been demonstrated that engineered secretion of ATXN10 from non-cachexia-inducing cells is sufficient to induce cachexia (muscle atrophy) phenotypes in cardiomyocytes, correlating with elevated ATXN10 serum levels in murine and human cancer cachexia models [62]. Interestingly, ATXN10, was upregulated 1.3-fold in LCs. Prompted by this observation, we adressed the question whether this increase is realted to a elevated expression of the corresponding gene or to strengthened protein stability. Our *ATXN10* transcript studies revealed 1.6-fold increase in MSS-patient derived cells compared to controls (Figure 8A) showing a forced gene expression upon SIL1-deficiency. Based on the tissue-specific pro-survival and pro-apoptotic properties of ATXN10, protein level were studied in serum, spleen, heart, kidney, spinal cord, quadriceps muscle and cerebellum. In serum samples derived from woozy animals, increased abundance of ATXN10 could be observed whereby the detected protein shows a band at 37 kDa in wildtype and a smaller one in woozy. One might assume that this results from cleavage as proteolytic cleavage of ataxin-7 modulating cellular toxicity has already been described [63]. Interestingly, ATXN10 level increase in neocerebellar PCs of wildtype mice showing a developmental expression pattern of this protein which might explain the early vulnerability of the SIL1-deficient neocerebellar PCs. However, surviving vestibulocerebellar PCs of woozy animals already show increase in young (6 weeks old animals) and this increase becomes more prominent in glial cells with disease progression (Figure 8B and 8C). Here, immunoblot studies showed apart from the 53 kDa band an addional band with a molecular weight over 60 kDa. Moreover, we found elevated ATXN10 level in spinal cord motor neurons (Figure 8b). Whereas no detrimental increase in the spleen and no increase in heart, and kidney could be observed, SIL1-deficient quadriceps muscle shows ATXN10 increase (Figure 8B), especially in degenerating fibres (Figure 8c). Here, immunoblot studies did not show additional protein bands apart from the expected protein sized 53 kDa (Figure 8B). In line with the de above mentioned functions, neuronal ATXN10 up-regulation might reflect a compensatory strategy to antagonize PC degeneration in SIL1-deficient cerebellum, whereas the increase in quadriceps muscle might promote fibre breakdown. Thus, ATXN10 expression in SIL1 mutant tissue contributes to selective vulnerability in this multisystemic disease.

To conclude, our study highlights that loss of functional SIL1 – even in apparently clinically non-affected tissues – results in morphological perturbations which are associated with altered protein expression. Proteomics revealed abnormal cytoskeletal and mitochondrial integrity, activation of antagonistic apoptotic and pro-survival mechanisms as well as altered expression of proteins necessary for function and maintenance of skeletal muscle fibers and neurons. Immunohistochemistry studies focusing on clinically non-affected (spleen and vestibulocerebellum) and affected (neocerebellum) cellular populations suggest that diverging expression of pro-survival and pro-apoptotic proteins as well as altered abundances of proteins important for neuronal integrity might be causative for selective vulnerability in MSS. Further studies highlighted ataxin-10 as a protein responsible for selective organ vulnerability in MSS. The combined results thus provide explanations for the selective organ damage upon the loss of the ubiquitous protein SIL1.

## MATERIALS AND METHEDS

### Patients, sample collection and preparation

Four MSS patients with genetically and biochemically proven *SIL1* mutations were included in this study (MSS24: p.V231_I232del; MSS32: p.G312R + p.F345fs; MSS33: p.256fs; MSS94: p.E101fs) [4]. All of them presented with the major clinical hallmarks of SIL1-related MSS. Lymphocytes of MSS patients and controls were obtained by peripheral venipuncture and immortalized using the protocol mentioned below. The recruitment and blood draw procedures were in accordance with the 1989 declaration of Helsinki and were approved by the Ethical committee of the University Hospital RWTH Aachen.

Patient lymphoblasts were collected from 10 ml heparin blood by making use of 40 ml of a buffer containing 150 mM NH_4_Cl, 10 mM KHCO_3_, and 125 mM EDTA for 10 minutes at room-temperature, and following centrifugation by 1,200 rpm for 10 minutes. Supernatant was discarded; the remaining cell pellet was re-suspended and incubated for 10 min in 50 mL of the same buffer. After another centrifugation step, the supernatant was discarded and the lymphoblast were incubated with 2 ml culturing medium of a B95-8 marmoset-monkey cell line for 1 hour at 37°C (5 % CO_2_). The mentioned medium containing the virus was perlocated before (45 μm filter, Millipore) in order to remove monkey cells. Infected lymphoblasts were afterwards transferred into cell culture flasks containing 3 ml RPMI 1640 medium with L-glutamine (PAA) (with 20 % FCS (Gibco), 1 % penicillin/streptomycin (PAA) and 0.05 % amphotericine) and 8 μl cyclosporine A (3 mg solved in 100 % ethanol and then supplemented with 60 μl Tween 20 (Sigma), 740 μl RPMI 1640 medium without FCS and 2 ml RPMI 1640 medium containing 20 % FCS)

### Cell culture

Culturing of the immortalized LCs was maintained at 37°C in a 5 % CO_2_ atmosphere. RPMI 1640 medium with L-glutamine (PAA) (with 20% FCS (Gibco), 1 % penicillin/streptomycin (PAA) and 0.05 % amphotericine) was used.

### Animals

All described procedures were approved by the UK Aachen Institutional Animal Care and Use Committee and conducted in compliance with the Guide for the Care and Use of Laboratory Animals. *Sil1* (located on murine chromosome 18) mutants were obtained from Jackson Laboratories (strain name: CXB5/By-Sil1wz/J; stock number 003777). Homozygously affected animals (woozy) as well as wild-type littermates were obtained by mating heterozygous males with heterozygous females. Litters were genotyped using the AccuPrime™ GC-Rich DNA Polymerase kit (Invitrogen) and 40 ng of DNA isolated from tail tissue utilizing a combination of three oligonucleotides (oIMR3762: GGTGTATCCAGGACTTGCTATGGTGG; oIMR3763: TCCACATTGCATCAGTCAGAACCG; oIMR3764: TCCGTTGCTCCGATGTCCGATACG). Afterwards, 8 μl of the PCR product (total volume 25 μl) were analyzed via electrophoresis (DNA was visualized uitilizing ethidium bromide). Mutant animals can be identified based on a ∼550 basepair (bp) sized product, wild-type animals based on a ∼450 bp product.

Fresh spleen samples of 12 mice and fresh cerebellar, spinal cord, kidney, heart and quadriceps muscle samples of 6 mice were directly fixed in 4 % buffered formalin for subsequent immunohistochemical studies (see below) and cerebellar as well as quadriceps muscle samples of 6 mice and spleen of four mice were directly lysed for subsequent immunoblot studies (see below).

### Protein lysate preparation and immunoblot studies

Murine tisse samples were transferred into lysis buffer containing 0.125 M Tris, pH 6.4, 4 % SDS, 10 % beta-mercaptoethanol, 10 % glycerin, 0.001 % bromophenol blue, 4 M urea and protease inhibitor mix, and lysed by sonication for 20 seconds (one second to one second pulses). Afterwards, homogenates were immediately cooled on ice for five minutes followed by heating for 15 minutes at 56°C. Sera have been mixed in a 1:1 ration with the above mentioned buffer and were afterwards also heated for 15 minutes at 56°C. Immortalized LCs were processed as described previously for immunoblot studies [23].

For each experiment, 10 μg protein of tissue homogenate (quantified with the BCA™ protein assay – reducing agent compatible kit (Pierce®) according to the manufacturer's instructions) were loaded on 10 % polyacrylamide gels and proteins have been separated for 120 minutes at 120 V followed by transfer on Immobilon®-P PVDF membrane (0.45 μm, Millipore®) with the help of the tank-blot technique over night at 5 V. After blocking with 1% casein buffer (1% casein; (Roche®) in Tris-buffered saline (TBS) and 0.1 % NP40 (Sigma®) as a 1:1 composition with maleic acid buffer which was composed of 100 mM maleic acid (Sigma®) and 150 mM NaCl) for two hours, membranes were washed three times in TBS (containing 0.1 % NP40) for 2 minutes, and incubated with different primary antibodies (Supplementary Table 3) diluted in 1% casein-buffer. PVDF-membranes were washed three times in TBS with 0.1 % NP40, and incubated for 1 hour with a horseradish peroxidase conjugated secondary goat anti-rabbit antibody (Sigma®) or goat anti-mouse antibody (Sigma®) at 1:25,000, dilutions, respectively. Afterwards, blots were washed three times for 10 minutes in TBS at any one time and signals were detected after application of enhanced chemiluminescence (ECL) HRP substrate (SuperSignal West Pico and SuperSignal West Femto; Pierce®) on CL-X Posure TM films (Thermo Scientific®).

Immunoprecipitation studies have been carried out as described previously [36].

### Immunohistochemistry

5 μm paraffin sections of murine spleen, kidney, hear, quadriceps muscle or cerebellum were placed on silane-coated slides, rehydrated and heat-unmasked, then blocked with PBS containing 2 % goat serum, and incubated overnight with the respective primary antibody (Supplementary Table 3) 1:100 diluted in blocking solution. Appropriate biotinylated secondary antibodies were used (1:200, Vektor Laboratories, USA). Cellular structures were counterstained with hematoxilin. For visualization, the DAB reaction (DAKO, USA) was used. Sections were viewed with the Eclipse 55i microscope (Nikon, Germany) and photographed using the Nikon digital sight.

### Preparation for transmission electron microscopy (TEM)

Immortalized lymphoblasts derived from five different MSS-patients as well as from healthy controls were collected by centrifugation at 1000 rpm for 5 minutes. Cells were washed in 0.1 M phosphate buffer and immediately fixed in 2.5 % glutardialdehyde buffer for 24 hours followed by buffering in 0.1 M phosphate buffer for 24 hours. Afterwards, cell pellets generated by centrifugation (as above) were embedded in 2 % agarose (at 60°C; Fluka #05073). Small blocks of embedded cells were sliced and post-fixed in 2.5 % glutardialdehyde for 24 hours and re-buffered in 0.1 M phosphate buffer for another 24 hours. Agarose blocks were subsequently incubated in 1% OsO4 (in 0.2 M phosphate buffer) for 3 hours, washed twice in distilled water and dehydrated using ascending alcohol concentrations (i.e. 25%, 35%, 50%, 70%, 85%, 95%, 100%; each step for 5 min). Afterwards, samples were incubated in propylenoxide followed by a 20 min incubation in a 1:1 mixture of epon (47.5% glycidether, 26.5% dodenylsuccinic acid anhydride, 24.5% methylnadic anhydride and 1.5% Tris (dimethylaminomethyl)phenol) and propylenoxide. Prepared samples were incubated in epoxy resin for 1 hour at RT followed by polymerization procedure (28°C for 8 hours, 80°C for 2.5 hours and finally at RT for 4 hours. Ultra-thin sections (70 nm) were prepared, mounted on grids for electron microscopy and examined using a Philips CM10 transmission electron microscope as described [18].

### Mass spectrometry

#### Cell lysis and carbamidomethylation

Approximately 2 mg of cells were lysed in 0.3 mL of 50 mM Tris-HCl, pH 7.8 - buffer containing - 150 mM sodium chloride (NaCl), 1% sodium dodecyl sulfate (SDS) and complete mini-EDTA free. Subsequently, 3 μL of benzonase (25 U/μL) and 2 mM magnesium chloride (MgCl_2_) were added to the lysates and incubated at 37°C for 30 min. Samples were centrifuged at 4°C and 14,000 g for 15 min and protein concentration was determined by bicinchoninic acid (BCA) assay according to the manufacturer's instructions (Pierce BCA Protein Assay Kit, Thermo Scientific). Cysteines were reduced with 10 mM dithiothreitol (DTT) at 56°C for 30 min and the free thiols were carbamidomethylated with 30 mM iodoacetamide (IAA) at RT for 30 min in the dark.

#### Sample preparation and proteolysis

Sample cleaning and proteolysis were based on filter aided sample preparation (FASP) protocol [21, 22]. Briefly, cell lysates corresponding to 100 μg of protein were diluted up to 400 μL with freshly prepared 8 M urea/100 mM Tris-HCl (pH 8.5) buffer [64]. Diluted samples were placed on the Microcon centrifugal devices (30 KDa cutoff) and were centrifuged at 13,800 g at RT for 20 min. All the following centrifugation steps were performed under similar conditions. To eliminate residual SDS, three washing steps were carried out using 100 μL of 8.0 M urea /100 mM Tris-HCl buffer, pH 8.5 and finally for the buffer exchange, the devices were washed thrice with 100 μL of 50 mM ammonium bicarbonate (NH_4_HCO_3_) buffer, pH 7.8. To the concentrated proteins, 100 μL of proteolysis buffer comprising trypsin (Promega) (1:50 w/w, enzyme to protein), 0.2 M guanidine hydrochloride (GuHCl), 2 mM calcium chloride (CaCl_2_) in 50 mM NH_4_HCO_3_, pH 7.8 were added and incubated at 37°C for 14 hours. The generated tryptic peptides were recovered by centrifugation with 50 μL of 50 mM NH_4_HCO_3_ followed by 50 μL of ultra-pure water. Finally, the peptides were acidified with 10% trifluoroacetic acid (TFA) to pH < 3 and the digests were quality controlled as described previously [65]. Acidified peptides were desalted with C18 solid phase extraction cartridges (SPEC, 4 mg, Varian) according to the manufacturer's instructions. The dried peptides were resolubilized in 0.5 M triethylammonium bicarbonate (TEAB) buffer, pH 8.5 and the peptide concentration was determined using NanoDrop 2000 UV-Vis spectrophotometer (Thermo Scientific).

#### Label free LC-MS/MS analysis

All eight samples (four healthy and four MSS patients, each 1 μg) were analyzed using an Ultimate 3000 nano RSLC system coupled to an Q Exactive mass spectrometer (both Thermo Scientific). Peptides were preconcentrated on a 75 μm x 2 cm C18 trapping column for 10 min using 0.1% TFA (v/v) with a flow rate of 20 μL/min followed by separation on a 75 μm x 50 cm C18 main column (both Pepmap, Thermo Scientific) with a 127 min LC gradient ranging from 3-42% of buffer B: 84% acetonitrile (ACN), 0.1% formic acid (FA) at a flow rate of 250 nL/min. The Q Exactive MS was operated in data-dependent acquisition (DDA) mode and MS survey scans were acquired from m/z 300 to 1,500 at a resolution of 70,000 using the polysiloxane ion at m/z 371.101236 as lock mass [66]. The fifteen (Top15) most intense signals were subjected to higher energy collisional dissociation (HCD) with a normalized collision energy (NCE) of 27% at a resolution of 17,500, taking into account a dynamic exclusion of 12 s. Automated gain control (AGC) target values were set to 3 × 10^6^ for MS and 5 × 10^4^ MS/MS. Maximum injection times (IT) were 120 ms and 250 ms, respectively.

#### Label free data analysis

Label free quantification of the acquired MS data was performed using the Progenesis LC-MS software from Nonlinear Dynamics (Newcastle upon Tyne, U.K.) version 4.1. MS data processing including alignment of raw data, selection of the reference LC-MS run and peak picking was done automatically by Progenesis. The features within retention time and m/z windows from 0-120 min and 300-1,500 m/z with charge states +2, +3, and +4 were considered for peptide statistics, analysis of variance (ANOVA) and principal component analysis (PCA). Spectra were exported as peak lists, searched against a concatenated target/decoy version of the human Uniprot database, (downloaded on 30^th^ of July 2012, containing 20,232 target sequences) using Mascot 2.4 (Matrix Science), OMSSA 2.1.9 and X!Tandem cyclone (version 2013.02.01.1) with the help of searchGUI 1.12.2 [67]. Trypsin was selected as enzyme with a maximum of two missed cleavage sites, carbamidomethylation of Cys was set as fixed and oxidation of Met was selected as variable modification. MS and MS/MS tolerances were set to 10 ppm and 0.02 Da, respectively. For combining the peptide and protein identifications obtained from the three search algorithms, we used our PeptideShaker software 0.22.0 [68]. The merged search results were filtered at a false discovery rate (FDR) of 1% and exported using the advanced PeptideShaker features that allow direct re-import of the quality-controlled data into Progenesis. To avoid unanticipated bias in quantification, peptide sequences containing Met, pyro-Glu and pyro-CMC (derived from X!Tandem 2^nd^ pass search) were excluded and only proteins that were quantified with ≥ 2 unique peptides were considered for further analysis.

#### iTRAQ 8-plex labeling and reversed phase fractionation at pH 6.0

For each sample, i.e. MSS and respective controls, 40 μg of tryptic peptides were labeled with iTRAQ 8-plex reagents according to the manufacturer's instructions. Afterwards, the samples were pooled and the multiplexed sample was dried completely under vacuum. Subsequently, the pellet was resolubilized in 0.5% TFA (pH < 3.0), desalted with C18 SPEC (15 mg, Varian) and the eluted peptides were dried in a SpeedVac. To reduce the sample complexity and to enhance proteome coverage, the sample was fractionated using reversed phase HPLC at pH 6.0. The dried multiplexed sample was resolubilized in buffer A (10 mM ammonium acetate, 0.4 mM FA, pH 6.0) and 50 μg were fractionated on a Zorbax 300SB-C18 column, 0.5 × 150 mm, 5 μm particle size column (Agilent) using an UltiMate 3000 HPLC (Thermo Scientific) with a binary buffer system; buffer A: 10 mM ammonium acetate, 0.4 mM FA, pH 6.0 and buffer B: 84% ACN in 10 mM ammonium acetate, 0.4 mM FA, pH 6.0. Peptides were loaded onto the column with buffer A at a flow rate of 12.5 μL/min and separation was carried out using the following gradient: 0-3% B in 10 min, 3-50% B in 65 min, 50-60% B in 5 min, 60-95% B in 5 min, 95% B hold for 5 min, 95%-3% B in 5 min and finally re-equilibrate the column with 3% B for 20 min. In total, 24 fractions were collected at 30 sec intervals from min 15 to 85 in a concatenation mode.

#### iTRAQ LC-MS/MS analysis

Each fraction was resolubilized in 30 μL of 0.1% TFA and 50% of the sample was analyzed using an Ultimate 3000 HPLC system coupled to an LTQ-Orbitrap Velos mass spectrometer (both Thermo Scientific) using the same buffers and gradient as described above. The MS was also operated in DDA mode wherein the five most intense precursor ions were subjected to HCD with a NCE of 47% and MS/MS scans were acquired in the Orbitrap at a resolution of 7,500, taking into account a dynamic exclusion of 30 s. Precursor isolation width was set as 2.0 m/z with an activation time of 0.2 ms. AGC target values for MS were set to 1 × 10^6^ and 1 × 10^5^ for MS/MS. Maximum IT were set to 50 ms and 200 ms, respectively.

#### iTRAQ data analysis

All iTRAQ raw data were processed simultaneously using the MudPIT option with Proteome Discoverer 1.3 (Thermo Scientific) and searched against a concatenated target/decoy version of the human Uniprot database. To maximize the number of peptide spectrum matches (PSMs), we included two different search algorithms (Mascot and SEQUEST) using the same set of parameters i.e., precursor and fragment ion tolerances of 10 ppm and 0.02 Da for MS and MS/MS, respectively; trypsin as enzyme with a maximum of 2 missed cleavages; carbamidomethylation of Cys, iTRAQ-8plex on N-terminus and Lys as fixed modifications; oxidation of Met as variable modifications. All data from Proteome Discoverer (PD) software 1.3 were exported with the following filter criteria: peptide spectrum matches (PSMs) with false discovery rate (FDR) < 1% (high confidence PeptideValidator setting), search engine rank 1 and proteins that were quantified with ≥ 2 unique peptides. Finally, in order to be consistent with the label free data, PSMs with oxidized Met were excluded from quantification.

#### Data analysis

Only non-modified (except for Cys-carbamidomethylation) and no Met containing peptides were considered. Data were normalized to compensate for systematic errors. For each protein, at least three quantitative values per condition were considered by removing potential outliers that might increase the standard deviation among the biological replicates and therefore interfere with T-test statistics. Finally, a T-test was conducted (2-sided, unpaired, heteroscedastic) and ratios MSS/control were calculated. For both datasets, iTRAQ and label free global median ratios (MDglobal) and standard deviations (SDglobal) were calculated by considering all corresponding protein ratios (MSS/controls). Only proteins with p-values <0.05 having ratios (MSS/controls) which were more than 2 SDglobal apart from the MDglobal were considered as regulated, namely iTRAQ ratios <0.75 or >1.32 and label free ratios of <0.64 and >1.56. To generate a list of potentially altered proteins from both quantitative analyses (Supplementary Table 1), following criteria were used to generate a final list of candidate proteins: *(i)* proteins with opposing quantitative values for iTRAQ and label free were removed (i.e. up vs down-regulation); *(ii)* proteins had to pass regulation criteria for at least one of the two datasets, label free or iTRAQ; *(iii)* however, proteins, which did not meet the p-value criteria (≤ 0.05), but were at least two-fold regulated between MSS and controls were considered further. For the obtained list of proteins, median ratios and T-test values were calculated for all combined label free and iTRAQ data points, considering an outlier removal. Thus, for the final list only proteins were considered that either passed the criteria for regulation, i.e. having MSS/control ratios more than 2 standard deviations apart from the mean (over all proteins and quantitative values), or showing at least a two-fold regulation, resulting in a total of 162 regulated proteins (Supplementary Table 2).

The mass spectrometry proteomics data have been deposited to the ProteomeXchange Consortium [69] via the PRIDE partner repository with the dataset identifier PXD003030.

The proteomic workflow performed in this study is visualized in Figure 1.

### Analysis of cellular fitness

MSS-derived LC and control LC were pooled respectively and seeded into 96-well plates (5,000 cells per well) with RPMI 1640 medium with L-glutamine (PAA) containing 20 % FCS (Gibco), 1 % penicillin/streptomycin (PAA) and 0.05 % amphotericine. H_2_O_2_ treatment of the cells (25 μM for 1 hour) was carried out in order to proof the hypothesis that efficient activation of pro-survival mechanisms in MSS-patient derived LC prevents these cells from accelerated cell death caused by additional stress burden. The cytotoxicity assay (Roche Diagnostics®) quantifies cytolysis based on the measurement of LDH activity released from damaged cells and was performed according to the manufacturer's protocol. Thereby, LDH release could be related to disruption of the plasma membrane. Measurement at 490 nm (reference 630 nm) was carried out via kinetic microplate reader (Infinite M200; Tecan) 30 minutes after application. Cell proliferation, reflecting mitochondrial activity and cellular viability, was determined using WST-1 reagent (Roche Diagnostics®) according to the manufacturer's protocol. WST-1 conversion to the red soluble formazan via mitochondrial succinate-tetrazolium reductase was measured at 450 nm (reference 630 nm) via kinetic microplate reader (Infinite M200; Tecan) 4 hours after application. Experiments were done in triplicate and repeated three times.

### *ATXN10* transcript studies

To investigate the expression of *ATXN10* in LCs derived from MSS patients and controls, total RNA was isolated using TRIzol® Reagent (Life Technologies). Here, aliquots of same cells used for the proteomic studies have been utilized and cDNA was synthesized from isolated RNA by incubation with random primers and SuperScript® III Reverse Transcriptase (Life Technologies) for 2 h at 37°C. Quantitative reverse transcription polymerase chain reaction (qRT-PCR) was performed SYBR® Green JumpStart™ Taq Ready Mix according to manufacturer's instructions (Sigma S4438). Hereby, following primers have been utilized: *ATXN10*_F: GAGCAGCGGAACCGAGAAAC, *ATXN10*_R: TCTGCAGGCAAGCTCAACAG.

Human 18S has been used as a control: 18S_F- GTAACCCGTTGAACCCCATT, 18S_R- CCATCCAATCGGTAGTAGCG. Three technical replicates of four different MSS-patient and control-derived LCs were examined with a corresponding no template control. The relative expression ratio (RER) was determined using the formula:

RER of *ATXN10* = 2^−ΔCt target(patient – control)^/2^−ΔCt ref(patient – control)^

## SUPPLEMENTARY TABLES







## Abbreviations

ACN: acetonitrile
BCA: bicinchoninic acid
GuHCl: guanidine hydrochloride
iTRAQ: isobaric tags for relative and absolute quantification
LC-MS/MS: liquid chromatography-tandem mass spectrometry
RT: room temperature
TFA: trifluoroacetic acid
Tris: tris(hydroxymethyl)aminomethane
