# Supplementary material for: In-depth phenotyping of lymphoblastoid cells suggests selective cellular vulnerability in Marinesco-Sjögren syndrome
Source: Oncotarget. 2017 Jul 28;8(40):68493–516. doi: 10.18632/oncotarget.19663 (PMC5620273; doi:10.18632/oncotarget.19663)
Supplement: Supplementary file 1 [file oncotarget-08-68493-s001.pdf]

In-depth phenotyping of lymphoblastoid cells suggests selective cellular vulnerability in Marinesco-Sjögren syndrome

SUPPLEMENTARY MATERIALS

Supplementary Table 1: List of all relatively quantified proteins from both analyses (label-free and iTRAQ experiments). In both datasets, the normalized abundance values (NAVs) of eight individual samples (four biological replicates each of MSS patients and respective controls) were used to calculate a final ratio of MSS/control and Student's T-Test (p-value < 0.05), respectively for each protein. For iTRAQ data, the NAVs were statistically treated in order to remove potential ouliers as described in the data analysis part of materials and methods section

See supplementary File: 1

Supplementary Table 2: Final list of significantly (see data analysis part of materials and methods section) differentially regulated proteins in MSS derived LCs obatined from both label-free and iTRAQ quantitative analyses

See supplementary File: 2

Supplementary Table 3: List of primary antibodies used for the immunohistochemistry and immunoblot studies of woozy and control mouse tissues and MSS-patient and control proposita derived LCs

Antigene	Source	Dilution immunohisto-chemistry	Dilution immuno-blotting	Company
Actin	rabbit	1:100	1:1000	BD Transduction Lab
Ataxin-10	rabbit	1:100	1:1000	Novus Biologicals
BiP	rabbit	1:100	1:1000	BD Transduction Lab
Calmodulin	rabbit	1:100	1:1000	Genetex
CDC123	rabbit	1:100	-	Genetex
Cytochrome C	rabbit	1:80	-	Merck Millipore
peif2α (Ser51)	rabbit	1:100	-	Abcam
GAPDH	rabbit	-	1:100	Genetex
GRP94	rabbit	1:100	1:100	Genetex
GRP170	rabbit	-	1:1000	Genetex
PHGDH	rabbit	1:80	1:750	Genetex
RAB11-FIP1A	rabbit	1:80	1:750	Abcam
SELH	rabbit	1:50	1:500	Provided by Prof. V. Gladyshev
Synuclein	mouse	1:100	1:500	Abcam
